# Cardiovascular safety of using non-steroidal anti-inflammatory drugs for gout: a Danish nationwide case-crossover study

**DOI:** 10.1007/s00296-024-05584-7

**Published:** 2024-04-06

**Authors:** Anne Bech-Drewes, Kasper Bonnesen, Ellen-Magrethe Hauge, Morten Schmidt

**Affiliations:** 1 Department of Clinical Epidemiology, Aarhus University Hospital, Aarhus, Denmark; 2 Department of Clinical Medicine, Aarhus University, Olof Palmes Allé 43, 8200 Aarhus, Denmark; 3 Department of Rheumatology, Aarhus University Hospital, Aarhus, Denmark; 4 Department of Cardiology, Gødstrup Regional Hospital, Aarhus, Denmark

**Keywords:** Cardiovascular disease, Gout, Non-steroidal anti-inflammatory drugs, Case-crossover study

## Abstract

Gout attacks are treated with uric-lowering and anti-inflammatory drugs. In patients with gout, non-steroidal anti-inflammatory drugs (NSAIDs) could be both cardiovascular beneficial, due to their anti-inflammatory actions, and cardiovascular hazardous, due to their prothrombotic, hypertensive, and proarrhythmic side effects. We, therefore, examined the risk of cardiovascular events associated with NSAID use in patients with gout. We conducted a nationwide, population-based case-crossover study of all Danes ≥ 18 years of age with first-time gout during 1997–2020, who experienced a cardiovascular event (myocardial infarction, ischemic stroke, congestive heart failure, atrial fibrillation/flutter, or cardiovascular death) (*n* = 59,150). The exposure was use of NSAIDs, overall and according to type (ibuprofen, naproxen, or diclofenac). We used the dates 300, 240, 180, and 120 before the outcome date as reference dates. We used the Mantel–Haenszel method to calculate odds ratios (ORs) with 95% confidence intervals (CIs) of the association between NSAID use and cardiovascular events. NSAID use was overall associated with 12% decreased odds of a cardiovascular event (OR = 0.88, 95% CI: 0.85–0.91). This decreased odds ratio was observed for the use of ibuprofen (OR = 0.92, 95% CI: 0.88–0.97) and naproxen (OR = 0.85, 95% CI: 0.74–0.97), but not for the use of diclofenac (OR = 0.97, 95% CI: 0.90–1.05). Overall, use of NSAIDs was associated with decreased odds of all the individual components of the composite outcome. NSAIDs were not associated with an increased cardiovascular event rate when used in gout patients. Ibuprofen and naproxen appeared to have better cardiovascular risk profiles than diclofenac.

**Supplementary Information:**

The online version contains supplementary material available at 10.1007/s00296-024-05584-7.

## Introduction

The prevalence of gout ranges from < 1% to 6.8% depending upon the population studied, making it the most common form of inflammatory arthritis globally [1]. Gout incidence has recently increased in developed countries [2–4]. For example, in Denmark, the annual incidence of gout increased by almost 80% between 1995 and 2015 [4]. Gout is characterized by the presence of monosodium urate crystals in joints or synovial fluid [3] as well as increased uric acid blood levels (hyperuricemia) [1]. Gout and hyperuricemia are considered cardiovascular risk factors [2]. Thus, a meta-analysis found an increased risk of myocardial infarction in patients with gout compared with patients without gout (relative risk = 1.22, 95% confidence interval (CI): 1.16–1.26) [5]. The increased cardiovascular risk in patients with gout might partly be explained by inflammation and oxidative stress, causing atherosclerotic plaque formation [6, 7].

The treatment of gout attacks aims at reducing inflammation, which is done with non-steroidal anti-inflammatory drugs (NSAIDs), low-dose colchicine, and/or glucocorticoids [8]. The treatment should be individualized with co-existing diseases considered [8, 9]. NSAID use has been associated with increased cardiovascular risks, even when used for a short period [10]. This increased risk is thought to be a complex altered equilibrium between cyclooxygenase (COX)-1 and COX-2 inhibition [11, 12]. However, in patients with inflammatory conditions, such as gout, the anti-inflammatory effects of NSAIDs might balance out their cardiovascular hazard. This risk–benefit balance is important for clinical decision-making. However, no study has examined NSAID-associated cardiovascular risks in patients with gout. Using patients as their own control in a case-crossover design, we, therefore, examined the cardiovascular event rate associated with NSAID use in patients with gout.

## Methods

### Settings

The Danish Health Service offers comprehensive tax-funded health care to both Danish citizens and legal residents. This includes free access to general practitioners and hospitals as well as partial reimbursement for prescribed medications, including NSAIDs [13]. Upon birth or immigration, all Danish citizens and legal residents receive a unique Civil Personal Registration number [13]. This system allows individual-level linkage between Danish registries as well as virtually complete long-term follow-up with accurate censoring at emigration or death [13].

### Study design

We conducted a nationwide, population-based case-crossover study of all patients with gout ≥ 18 years of age from January 1, 1996, to December 31, 2020, who experienced a cardiovascular event [14]. We identified patients with gout through either a primary, in- or outpatient gout diagnosis or through a filling of an allopurinol prescription. The gout diagnoses were identified via the Danish National Patient Registry [15]. This registry contains nationwide information on non-psychiatric inpatient contacts since 1977, and on psychiatric inpatient as well as all outpatient and emergency contacts since 1995 [15]. Gout patients who are solely treated by their general practitioner, however, do not have their gout registered in the Danish National Patient Registry [15]. We, therefore, also identified patients with gout using filled prescriptions for allopurinol from the Danish National Prescription Registry [16]. Allopurinol has only few FDA-approved indications besides gout [17], including prevention of recurrent calcium nephrolithiasis in patients with hyperuricosuria, and preventing of tumor lysis syndrome [17]. The Danish National Prescription Registry contains nationwide information on filled prescriptions from all community pharmacies since 1995 [16]. Supplementary Table S1 presents all codes used in the study.

### Exposure

The exposures were the use of NSAIDs, both overall and according to type (ibuprofen, naproxen, or diclofenac) identified via filled prescriptions from the Danish National Prescription Registry [16]. Because this registry does not contain information on the length of treatment or daily dose, we defined NSAID exposure as two tablets per day no matter the dose [18]. If a patient filled a new prescription for the same NSAID within a use period plus a 14-day grace period, the use period was extended by the number of days provided by the new prescription. If there went more than 14 days after a use period and a new prescription was filled, the new period of use would start on the day the new prescription. The grace period was added to the use period in both situations (*i.e.*, prospective filling of gaps) [19].

### Outcome

The primary outcome was a cardiovascular event defined as a composite of atrial fibrillation/flutter, congestive heart failure, myocardial infarction, ischemic stroke, and cardiovascular death [20]. The secondary outcomes included the individual cardiovascular diseases [20]. Cardiovascular events were identified from the Danish National Patient Registry and the main underlying cause of death from the Danish Register of Causes of Death [15]. Since 1970, The Danish Register of Causes of Death has contained information on the primary underlying cause and any potential contributory cause(s) of deaths [21].

### Covariables

The Comorbidities were identified using in- and outpatient medical history from the Danish National Patient Registry in the 5 years preceding the occurrence of a cardiovascular event. Every hospital discharge from 1977 and each outpatient clinic visit from 1995 onwards is documented in the registry, assigning one primary diagnosis and possibly multiple secondary discharge diagnoses, categorizing according to the International Classification of Diseases, Eighth Revision until the end of 1993 and thereafter the Tenth Revision [15]. Generally, the positive predictive values for most of the employed comorbidities were found to be high (> 90) [22]. Comorbidity burden was categorized according to the Danish Index for Acute Myocardial Infarction (DANCAMI) as no (score: 0), low (score: 1–3), moderate (score: 4–5), or severe (score: ≥ 6) [23].

Comedication use was defined as a filled prescription registered in the Danish National Prescription Registry within 90 days prior to the occurrence of a cardiovascular event. Comedications, such as statins, are employed as an indirect measure of identifying diagnoses like hyperlipidemia. Importantly, the self-control design controls for time-stable confounders by design. Thus, we presume consistent eating and exercise patterns between the event date and the earliest reference date (i.e., 300 days). As patients with cancer might receive allopurinol to prevent tumor lysis syndrome, we defined individuals as having cancer-related gout if they had either received a cancer diagnosis or filled a prescription for an antineoplastic agent in the 5 years before their gout diagnosis. Table 1 contains the identified comorbidities and comedications.

**Table 1:** Characteristics of patients with gout experiencing a cardiovascular event, Denmark, 1996–2020

Characteristics	Number (%)
Total	59,150 (100)
Male sex	40,443 (68)
Age in years, median (interquartile range)	72 (62–79)
≤ 65	19,260 (33)
65–74	16,859 (29)
≥ 75	23,031 (39)
Comorbidity burden†	
None (score: 0)	16,925 (29)
Low (score: 1–3)	16,814 (28)
Moderate (score: 4–5)	9,550 (16)
Severe (score: ≥ 6)	15,861 (27)
Comorbidities	
Myocardial infarction	5,188 (8.8)
Ischemic stroke	1,916 (3.2)
Venous thromboembolism	1,602 (2.7)
Chronic kidney disease	5,439 (9.2)
Diabetes	9,619 (16)
Chronic pulmonary disease	8,950 (15)
Hypertension	22,902 (38)
Obesity	2,936 (5.0)
Hyperthyroidism	894 (1.5)
Inflammatory rheumatic disease	11,526 (19)
Degenerative rheumatic disease	10,575 (18)
Soft tissue disorders	3,626 (6.1)
Osteoporosis	1,289 (2.2)
Headache	220 (0.37)
Alcoholism	1,026 (1.7)
Diseases of the liver	732 (1.2)
Comedication	
Acetylcholinesterase inhibitors	13,593 (23)
Angiotensin II receptor blockers	4,876 (8.2)
Beta-blockers	18,721 (32)
Calcium channel blockers	10,497 (18)
Diuretics	30,893 (52)
Nitrates	6,006 (10)
Statins	12,615 (21)
Anti-coagulants	9,226 (16)
Anti-platelets	17,186 (29)
Selective serotonin reuptake inhibitors	2,707 (4.6)
Systemic glucocorticoids	5,140 (8.7)
Type of gout	
Cancer related‡	5,290 (8.9)
Not cancer related	53,860 (91)

Cardiovascular event is defined as composite of myocardial infarction, ischemic stroke, congestive heart failure, atrial fibrillation/ flutter, and cardiovascular death

^†^Defined according to the Danish Comorbidity Index for Acute Myocardial Infarction (DANCAMI)

^‡^Defined by the Anatomical Therapeutic Chemical Classification (see Supplementary Table S1) or the primary, in-/outpatient International Classification of Diseases 10th edition code DC (cancers) in the five years before gout

### Statistical analyses

**Fig. 1:**
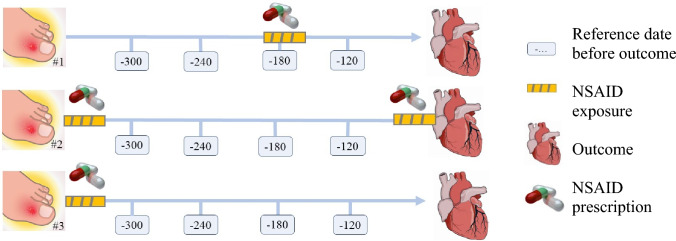
The case-crossover study design. The self-controlled design compares, among individuals experiencing an outcome, the number of individuals exposed to NSAID use at the outcome date, but not at any reference date, with the number of individuals exposed at a reference date, but not at the outcome date. Patient #1 is exposed at the second reference point from the outcome. Patient #2 is exposed at the outcome date. Patient #3 is not exposed at any reference day or outcome date and is dropped from the analysis. Using Mantel–Haenszel method, the odds ratios of being exposed on the outcome date vs. the reference date is calculated by dividing the first pattern with the second pattern

We present continuous variables as medians with interquartile ranges and categorical variables as numbers with percentages. In our self-controlled design, we compared, among individuals expiring a cardiovascular event, the number of individuals using NSAIDs at the day of the event but not at a reference date with the number of individuals using NSAIDs at a reference date, but not at the event date (Fig. 1) [18, 24]. We used the Mantel–Haenszel method to calculate odds ratios (ORs) with 95% confidence intervals (CIs) of the association between NSAID use and a cardiovascular event [24, 25]. As previously applied, we used the dates 300, 240, 180, and 120 before the outcome date as reference dates [18]. By using fixed outcome and reference dates, rather than fixed windows stacked backwards in time from the outcome and reference dates, we allowed for flexibility when assigning the exposure length after a filled prescription. By having a 120-day gap between the last reference date and the outcome date, compared with only a 60-day gap between the individual reference dates, we secured a 60-day washout window [26]. As everyone serves as their own control, the self-control design controls for confounding by time-stable variables, such as genetics, by design.

We performed the analyses within subgroups according to sex, age, comorbidity burden, cancer-related gout, and whether gout was identified via a diagnosis or via a filled allopurinol prescription. All analyses were performed using Stata version 17.0 (StataCorp LLC, College Station, TX, USA).

## Results

### Patient characteristics

**Fig. 2:**
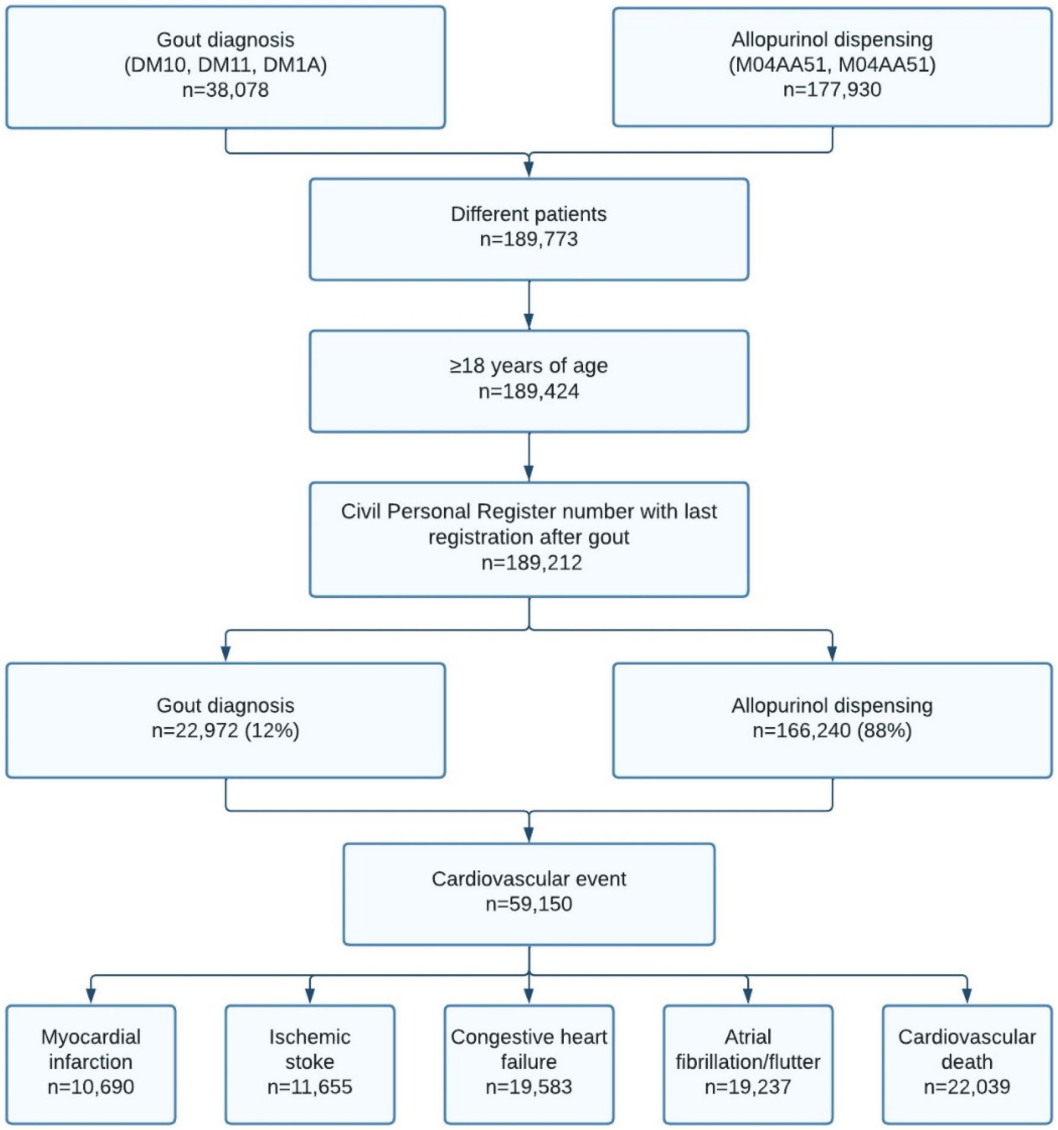
Flowchart of the study populations, 1996–2020

We identified 59,150 individuals with either a first-time gout diagnosis (12%) or a filled allopurinol prescription (88%) who experienced a cardiovascular event during follow-up from 1996 to 2020 (Fig. 2). The time from gout diagnosis to a cardiovascular event differed whether gout was identified via a diagnosis (median = 924 days, interquartile range: 279–2167) or via a filled allopurinol prescription (median = 1373 days, interquartile range: 462–3032). The average number of filled allopurinol prescriptions in the study period were 13 (median = 5, interquartile range: 2–16). Among the included patients with gout, 68% were males, their median age was 72 years (interquartile range: 62–79), 29% had no comorbidity burden, 27% had a severe comorbidity burden and 8.9% had gout in relation to cancer (Table 1). The most common comorbidities were hypertension (38%), other inflammatory diseases (19%), degenerative rheumatic disease (18%), diabetes (16%), chronic pulmonary disease (15%) and chronic kidney disease (9%). The most used drugs were diuretics (52%), beta-blockers (32%), and antiplatelets (29%).

### Cardiovascular risks

**Fig. 3:**
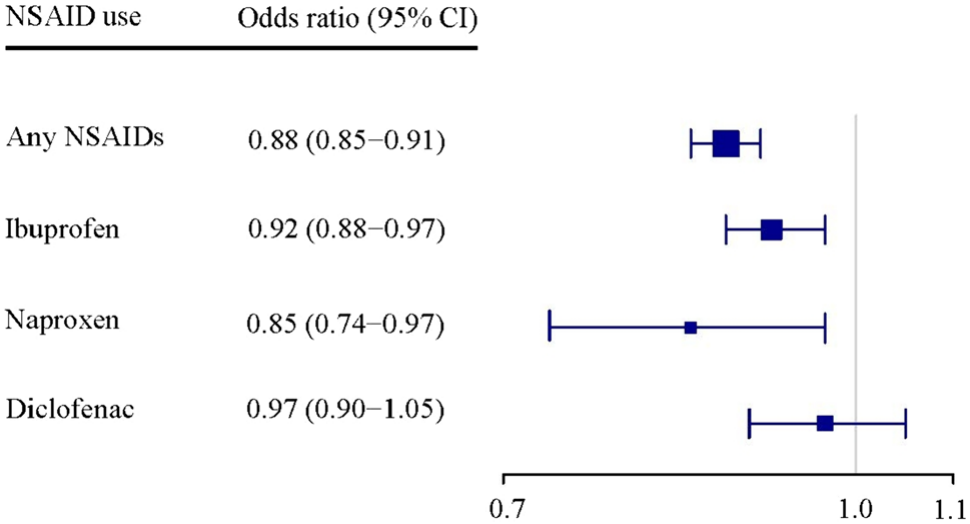
Non-steroidal anti-inflammatory drugs (NSAIDs) and adverse cardiovascular events in patients with gout. Number of disconcordant pairs: 13,564 for any NSAID, 7,658 for ibuprofen, 988 for naproxen, 3,152 for diclofenac. Adverse cardiovascular event is a composite of myocardial infarction, ischemic stroke, congestive heart failure, atrial fibrillation or flutter, and cardiovascular death

Compared with when not using NSAIDs, slightly decreased odds of a cardiovascular event were found for use of NSAIDs overall (OR = 0.88, 95% CI: 0.85–0.91), ibuprofen (OR = 0.92, 95% CI: 0.88–0.97), and naproxen (OR = 0.85, CI: 0.74–0.97) (Fig. 3). Use of diclofenac was not associated with a cardiovascular event (OR = 0.97, 95% CI: 0.90–1.05).

Compared with when not using NSAIDs, use of any NSAID was associated with decreased odds of all secondary outcomes, *i.e.*, myocardial infarction (OR = 0.91, 95% CI: 0.84–0.99), ischemic stroke (OR = 0.88, 95% CI: 0.81–0.95), congestive heart failure (OR = 0.89, 95% CI: 0.83–0.94), atrial fibrillation/flutter (OR = 0.80, 95% CI: 0.75–0.85), and cardiovascular death (OR = 0.60, 95% CI: 0.56–0.65) (Table 2). Regarding the type of NSAID, compared with when not using NSAIDs, ibuprofen use was associated with a decreased odds of ischemic stroke (OR = 0.86, 95% CI: 0.77–0.96), atrial fibrillation/flutter (OR = 0.82, 95% CI: 0.75–0.90), and cardiovascular death (OR = 0.67, 95% CI: 0.61–0.74). Naproxen use was associated with a decreased odds of cardiovascular death (OR = 0.53, 95% CI: 0.40–0.71). Diclofenac use was associated with a decreased odds of atrial fibrillation/flutter (OR = 0.83, 95% CI: 0.71–0.96) and cardiovascular death (OR = 0.87, 95% CI: 0.76–0.99) (Table 2).

**Table 2:** Non-steroidal anti-inflammatory drugs (NSAIDs) and adverse cardiovascular events in patients with gout

Outcome	Odds ratio (95% confidence interval) comparing use with non-use			
	Any NSAID	Ibuprofen	Naproxen	Diclofenac
Myocardial infarction	0.91 (0.84–0.99)	0.98 (0.88–1.10)	0.73 (0.52–1.00)	0.96 (0.81–1.14)
Ischemic stroke	0.88 (0.81–0.95)	0.86 (0.77–0.96)	0.90 (0.67–1.23)	1.04 (0.88–1.23)
Congestive heart failure	0.89 (0.83–0.94)	0.94 (0.86–1.02)	0.81 (0.63–1.04)	0.89 (0.77–1.02)
Atrial fibrillation/flutter	0.80 (0.75–0.85)	0.82 (0.75–0.90)	0.85 (0.66–1.11)	0.83 (0.71–0.96)
Cardiovascular death	0.60 (0.56–0.65)	0.67 (0.61–0.74)	0.53 (0.40–0.71)	0.87 (0.76–0.99)

### Subgroup analyses

The results remained robust after stratifying by sex (Table 3). Interpretation of the age- and comorbidity-stratified results were limited due to low precision (Table 3). Compared with a period when not using NSAIDs, use of any NSAIDs was associated with a decreased risk of cardiovascular events when restricting to patients without previous cancer (OR = 0.87, 95% CI: 0.84–0.90) (Table 3). The results did not differ notably according to whether gout was defined via a hospital diagnosis or via a filled allopurinol prescription (Table 3).

**Table 3:** Non-steroidal anti-inflammatory drugs (NSAIDs) and adverse cardiovascular events in patients with gout, according to the subgroups

Patient subgroup	Odds ratio (95% confidence interval) comparing use with non-use			
	Any NSAID	Ibuprofen	Naproxen	Diclofenac
Sex				
Male	0.88 (0.84–0.92)	0.92 (0.87–0.97)	0.85 (0.72–1.00)	0.85 (0.72–1.00)
Female	0.88 (0.83–0.94)	0.93 (0.85–1.02)	0.85 (0.65–1.10)	0.85 (0.65–1.10)
Age group				
≤ 64	0.86 (0.81–0.91)	0.90 (0.90–0.97)	0.90 (0.90–1.11)	0.90 (0.90–1.04)
65–74	0.78 (0.73–0.84)	0.83 (0.75–0.91)	0.79 (0.60–1.04)	0.86 (0.75–1.00)
≥ 75	0.98 (0.93–1.04)	1.03 (0.95–1.12)	0.83 (0.66–1.05)	1.12 (1.00–1.25)
Comorbidity burden*				
None (score: 0)	0.88 (0.82–0.93)	0.92 (0.85–1.00)	0.80 (0.62–1.02)	0.92 (0.80–1.05)
Low (score: 1–3)	0.90 (0.84–0.96)	0.96 (0.88–1.05)	0.85 (0.66–1.09)	0.99 (0.86–1.14)
Moderate (score: 4–5)	0.82 (0.75–0.89)	0.82 (0.73–0.93)	0.76 (0.52–1.11)	1.09 (0.91–1.30)
Severe (score: ≥ 6)	0.91 (0.84–0.98)	0.95 (0.86–1.05)	0.98 (0.74–1.30)	0.96 (0.82–1.12)
Type of gout†				
Cancer related	0.99 (0.87–1.13)	1.09 (0.91–1.29)	0.69 (0.39–1.22)	0.92 (0.68–1.24)
Not cancer related	0.87 (0.84–0.90)	0.91 (0.87–0.96)	0.86 (0.74–0.99)	0.98 (0.90–1.05)
Assessment of gout				
Gout diagnosis	0.95 (0.86–1.05)	1.06 (0.94–1.20)	0.71 (0.47–1.06)	1.05 (0.85–1.30)
Allopurinol prescription	0.87 (0.84–0.90)	0.90 (0.86–0.95)	0.87 (0.75–1.00)	0.96 (0.89–1.04)

^*^Comorbidity burden according to the DANish Comorbidity index for Acute Myocardial Infarction (DANCAMI) scores of 0 (none), 1–3 (low), 4–5 (moderate), and ≥ 6 (severe) [23]

^†^Defined by receiving a cancer diagnosis or filled a prescription for an antineoplastic agent in the five years before their gout diagnosis

## Discussion

This study examined the cardiovascular risks associated with NSAID use among patients with a first-time gout attack. Our findings indicate that the overall use of NSAIDs in patients with gout was not associated with an increased risk of adverse cardiovascular events. This finding supports the current clinical practice of treating gout with NSAIDs. In fact, the use of particularly ibuprofen and naproxen were linked to a slightly reduced risk of adverse cardiovascular events compared with non-use and, therefore, seem preferable to diclofenac. These results remained robust for various cardiovascular outcomes.

### Previous literature

Chronic inflammation has been associated with up to a 70% increased risk of cardiometabolic disorders [27]. Gout is characterized by low-grade inflammation, which leads to elevated levels of reactive oxygen species, proinflammatory cytokines, endothelial dysfunction, formation of neutrophil extracellular traps, and platelet hyperactivity. All of these factors may contribute to the development of atherosclerosis [28]. Additionally, hyperuricemia in patients with gout can cause oxidative stress, further damaging the endothelia [28]. Accordingly, a self-controlled study found that individuals with gout, who experienced a cardiovascular event, had twice the likelihood of having a gout flare in the days leading up the event, indicating that gout flares are linked to a temporary rise in cardiovascular risk [29]. Moreover, patients with inflammatory diseases are at higher cardiovascular risk due to the presence of traditional risk factors such as sex, hypertension, diabetes, smoking, and others [28].

Both randomized controlled trials and observational studies have provided evidence that NSAIDs increase the risk of cardiovascular events in general even for short treatment periods and low doses [11, 12, 18, 30], but data have been lacking in gout patients whose chronic inflammation may alter the risk–benefit balance. We did not find an increased odds of cardiovascular events when using NSAIDs in patients with gout. While the exact mechanism of the neutral or slightly beneficial effect of NSAID use in patients with gout remains unknown, we speculate that it relates to the anti-inflammatory properties of NSAIDs [6, 7]. Blocking a central mediator in the inflammation of gout patients (NLRP3 inflammasome) prevents cardiovascular events by activating the cytokine interleukin-1β, which affects the development of atherothrombotic plaques [27, 31]. Furthermore, it has been shown that, if left untreated, gout is associated with a two-fold increased risk of coronary heart disease, but treated (allopurinol, colchicine, sulfinpyrazone) the prognosis is comparable to the general population [32]. These results correspond with the decreased cardiovascular risk associated with NSAID use at time of the event found in our study. Finally, our findings also support previous literature suggesting a more favorable cardiovascular risk profile for the nonselective NSAIDs ibuprofen and naproxen than for the COX-2 selective diclofenac [12].

### Strengths and limitations

The large cohort of patients with frequent use of NSAIDs increased precision and allowed for examinations of associations on individual NSAIDs.

The free access to health care and the virtually complete long-term follow-up reduced the risk of selection bias from selective inclusion of health insurance systems, specific hospitals, or age groups. Using a filled allopurinol prescription, it was possible to locate further cases not diagnosed at a hospital. However, this may also present limitations as it is possible that we have included patients who were treated with allopurinol for other conditions than gout such as tumor lysis syndrome. However, the number treated with allopurinol for such diseases is likely small due to few indications [17], resulting in only a few non-gout patients included in the study. Even though the study by design only included patients with gout who suffered a cardiovascular event, the study results are considered relevant for gout attacks in general.

As the Danish National Prescription Registry lacks information on in-hospital NSAID use and over-the-counter NSAID sales, we might have misclassified in-hospital NSAID users or over-the-counter ibuprofen users as non-users [33]. However, in-hospital NSAID use is limited, and during the study period, ibuprofen was the only NSAID available over the counter, constituting approximately 15–25% of total NSAID sales [33]. This potential misclassification of NSAID, therefore, cannot noteworthy bias effect estimates of the association between NSAID use and cardiovascular events [34]. Coding of the gout diagnosis has not been validated in the Danish National Patient Registry [15]. However, we aligned the codes with recommendations from rheumatologists about current and previous coding practices of gout in the hospital setting. To increase completeness, we also identified patients through filled allopurinol prescriptions, which have few indications besides gout*.* The cardiovascular diagnosis has been validated with a positive predictive value of around 90% for myocardial infarction and ischemic stroke, 80% for heart failure, and 95% for atrial fibrillation [35, 36]. Mortality data are complete and accurate [13].

Even though the self-controlled design is robust to confounding by time-stable co-variables, it may be influenced by time-varying co-variables such as disease severity. The design is further prone to confounding-by-indication, if the patients would use NSAIDs for conditions associated with the outcome (for example using NSAIDs to treat angina). However, the consistency in the results of the individual outcomes indicates that this cannot explain the finding of no increased risk overall.

## Conclusion

In patients with gout, we found that NSAID use was not associated with increased cardiovascular risks at the time of a first gout attack. Furthermore, the use of ibuprofen or naproxen seemed to be associated with a lower cardiovascular risk than diclofenac. This information may be taken into consideration when selecting NSAIDs for patients with gout.

### Supplementary Information

Below is the link to the electronic supplementary material.

Supplementary file1 (DOCX 23 kb)

## Data Availability

Not allowed.

## Declarations

### Related congress abstract publications

Abstract for poster presentation at ESC 2023: AM Drewes, K Bonnesen, EM Hauge, M Schmidt. Cardiovascular safety of non-steroidal anti-inflammatory drug use for gout: A Danish nationwide case-crossover study. European Heart Journal, volume 44, issue supplement_2, November 2023, ehad655.2789, http://doi.org/10.1093/eurheartj/ehad655.2789. Abstract 545 for poster presentation at ICPE 2023: AM Drewes, M Schmidt, K Bonnesen, EM Hauge. Cardiovascular safety of non-steroidal anti-inflammatory drug use for gout: A Danish nationwide case-crossover study. Pharmacoepidemiology and Drug Safety, volume 32, issue S1, p. 3–612, 12 October 2023, https://doi.org/10.1002/pds.5687.

### Previously online posted pre-publication

The manuscript and the data are not submitted or published elsewhere.

### Transparency declaration

The authors affirm that the manuscript is an accurate, honest and transparent account of the study being reported. No aspect of the study has been omitted. Any discrepancies from the study as planned have been explained.

### Patient involvement statement

No patient involvement.
