# Supplementary material for: Cardiovascular safety of using non-steroidal anti-inflammatory drugs for gout: a Danish nationwide case-crossover study
Source: Rheumatol Int. 2024 Apr 6;44(6):1061–9. doi: 10.1007/s00296-024-05584-7 (PMC11108875; doi:10.1007/s00296-024-05584-7)
Supplement: Supplementary file 1 — Supplementary file1 (DOCX 23 kb) [file 296_2024_5584_MOESM1_ESM.docx]

**Supplementary**

**Supplementary Table S1.** Codes used for classification of exposure, outcome and comorbidity.

| **Classification** | **Variable** | **Codes** |
| --- | --- | --- |
| **Anatomical Therapeutic Chemical Classification** | | |
|  | Any NSAID | M01A |
|  | Ibuprofen | M01AE0, M01AE51, M02AA13 |
|  | Naproxen | M01AE02, M01AE52, M01AE56, M02AA12 |
|  | Diclofenac | M01AB05, M01AB55, M02AA15 |
|  | Antineoplastic agents | L01 |
| **Comedication** |  |  |
|  | ACE inhibitors | C09AA, C02EA |
|  | Angiotensin 2 Receptor Blockers | C09CA, C02EX01 |
|  | Beta-blockers | C07 |
|  | Calcium channel blockers | C08 |
|  | Diuretics | C03 and C02DF01 |
|  | Nitrates | C01DA |
|  | Statins | C10AA, C10B, B04AB |
|  | Anticoagulants | B01AA03, B01AA04, B01AB, B04AX04, B01AE, B01AX02, B01AX03, B01AF, B01AX excluding B01AX02, B01AX03, B01AX06 |
|  | Antiplatelet drugs | B01AC06, N02BA01, B01AC04, B01AC07, C01DA01, B01AC30 |
|  | SSRIs | N06AB |
|  | Systemic glucocorticoids | H02AB |
|  | Antidiabetics | A10 |
| **International Classification  of Diseases codes** | | |
|  | Myocardial infarction | ICD-8: 410; ICD-10: DI21 |
|  |  |  |
|  | Ischemic stroke | ICD-8: 433; ICD-10: DI63, DI64 |
|  | Congestive heart failure | DI110, DI130, DI132, DI420, DI426, DI427, DI428, DI429, DI500, DI501, DI502, DI503, DI508, DI509 |
|  | Atrial fibrillation/flutter | DI48 |
|  |  |  |
|  | Cardiac death | I0-152 |
| **Comorbidities** | | |
|  | Venous thromboembolism | ICD-8: 451.00; ICD-10: I80.1-3, I26 |
|  | Chronic kidney disease | ICD-8: 249.02, 250.02, 753.10-753.19, 582, 583, 584, 590.09, 593.20, 792; ICD-10: E10.2, E11.2, E14.2, N03, N05, N11.0, N14, N16, N18-N19, N26.9, Q61.1-Q61.4 |
|  | Cancer | ICD-8: 140-207; ICD-10: C00-C96 |
|  | Diabetes mellitus | ICD-8: 249-250; ICD-10: E10-E14, O24 (except O24.4), H36.0; ATC: A10B, A10A |
|  | COPD | ICD-8: 491-492; ICD-10: J41-44; ATC: R03 |
|  | Hypertension | ICD-8: 400-404; ICD-10: I10-I15 or combination of ≥2 of the following classes: α adrenergic blockers (C02A, C02B, C02C), non-loop diuretics (C02DA, C02L, C03A, C03B, C03D, C03E, C03X, C07C, C07D, C08G, C09BA, C09DA, C09XA52), vasodilators (C02DB, C02DD, C02DG, C04, C05), beta blockers (C07), calcium channel blockers (C07F, C08, C09BB, C09DB), and renin-angiotensin system inhibitors (C09). |
|  | Obesity | ICD-8: 277; ICD-10: E65-E68 |
|  | Hyperthyroidism | ICD-8: 242; ICD-10: E05 |
|  | Inflammatory rheumatic disease | ICD-8: 446, 712-714, 715-716, 135.99; ICD-10: M02-03, M05-14, M30–M36, M45-46. |
|  | Reactive arthritis | ICD-8: 714; ICD-10: M02-03 |
|  | Inflammatory polyarthritis | ICD-8: 712.09-712.39; ICD-10: M05-14 |
|  | Inflammatory spondylopathies | ICD-8: 712.49; ICD-10: M45-46 |
|  | Systemic connective tissue disease | ICD-8: 716, 734, 446, 135.99; ICD-10: M30–M36 |
|  | Degenerative rheumatic disease | ICD-8: 713, 725, 728; ICD-10: M15-25, M47-54 |
|  | Osteoarthritis | ICD-8: 713; ICD-10: M15-19 |
|  | Other joint disorders, including arthralgia | M20-25 |
|  | Spondylosis and other spondylopathies | M47-49 |
|  | Intervertebral disc disorders | M50-51 |
|  | Dorsalgia | ICD-8: 725, 728; M53-54 |
|  | Soft tissue disorders | ICD-8: 716-717; ICD-10: M65-790 |
|  | Disorders of synovium and tendons | ICD-10: M650-689 |
|  | Fibromyalgia | ICD-10: M79.7 |
|  | Other soft tissue disorders | ICD-10: M700-791 |
|  | Osteoporosis | ICD-8: 723.09; ICD-10: M80-M82 |
|  | Headache | ICD-8: 791.99; ICD-10: G43-44 |
|  | Alcoholism | ICD-8: 303. ICD-10: F10 |
|  | Diseases of liver | ICD-8:570-577, ICD-10: K70-K77 |
| **The DANish Comorbidity  index for Acute Myocardial Infarction (DANCAMI)** | | |
|  | Rheumatic disease |  |
|  | Connective tissue disease | M05, M06, M08, M09, M30–M36, D86 |
|  | Bone disorder | M80–M83, M85, M86.3–M86.6, M88 |
|  | Cardiovascular diseases |  |
|  | Heart failure | I50, I11.0, I13.0, I13.2 |
|  | Cardiomyopathy | I25.5, I42, I43 |
|  | Myocardial infarction (index disease) | I21 |
|  | Stroke | I60, I61, I63, I64 |
|  | Hypertension | I10–I13, I15, I67.4 |
|  | Stable angina pectoris | I20.1, I20.8, I20.9, I25.1, I25.9 |
|  | Intermittent arterial claudication | I73.9 |
|  | Aortic disease | I71 |
|  | Deep vein thrombosis in the lower limb | I80.1-I80.3 |
|  | Pulmonary embolism | I26.0, I26.9 |
|  | Atrial fibrillation/flutter | I48 |
|  | Heart block (atrioventricular block, left bundle-branch block, fascicular block) | 144, I45 |
|  | Ventricular tachycardia | I47.2 |
|  | Valvular heart disease | I05–I09, I34–I39 |
|  | Pulmonary disease |  |
|  | Chronic pulmonary disease | J40–J47, J60–J67, J68.4, J70.1, J70.3, J84.1, J92.0, J96.1, J98.2, J98.3^#^ |
|  | Kidney disease |  |
|  | Chronic kidney disease | E10.2, E11.2, E14.2, I12, I13, N03, N05, N11.0, N14, N16, N18, N19, N26.9, Q61.1–Q61.4, Z99.2 |
|  | Hematologic disease |  |
|  | Coagulopathy | D55–D61, D63, D64, D66–D72, D74–D77 |
|  | Immune system disorder | D80–D84, D89 |
|  | Nutritional anemia | D50–D53 |
|  | HIV | B21–B24 |
|  | Cancer |  |
|  | Low-risk cancer | C00–C12, C14, C17–C21, C30–C32, C37–C44, C46–C76, C80–C91, C92.1, C93, C94, C96, C97 |
|  | High-risk cancer | C13, C15, C16, C22–C26, C33, C34, C45, C77–C79, C92.0, C92.3–C92.9, C95 |
|  | Endocrine disease |  |
|  | Obesity | E65–E68 |
|  | Endocrine disorder (not diabetes) | E01–E03, E05, E06.2, E06.3, E06.5, E07, E20–27, E31, E32, E34.8, E34.9 |
|  | Diabetes uncomplicated | E10.0, E10.1, E10.9, E11.0, E11.1, E11.9 E12.0, E12.1, E12.9 E13.0, E13.1, E13.9 E14.0, E14.1, E14.9 |
|  | Diabetes with end-organ damage | E10.2–E10.8, E11.2–E11.8, E12.2–E12.8, E13.2–E13.8, E14.2–E14.8, H36.0 |
|  | Gastrointestinal disease |  |
|  | Moderate to severe liver disease | B15.0, B16.0, B16.2, B19.0, K70.4, K72, K76.6, I85 |
|  | Inflammatory bowel disease | K50, K51 |
|  | Chronic pancreatitis | K86.0, K86.1 |
|  | Ulcer disease | K22.1, K25–K28 |
|  | Mild liver disease | B18, K70.1–K70.3, K70.9, K71, K73, K74, K76.0 |
|  | Neurologic disease |  |
|  | Epilepsy | G40, G41 |
|  | Neurodegenerative disorder | G10–G13, G20-23, G25.5, G31.2, G31.8, G31.9, G35–G37, G90, G93.4 |
|  | Dementia | F00–F03, F05.1, G30 |
|  | Transient ischemic attack | G45 |
|  | Hemiplegia | G81, G82 |
|  | Psychiatric disease |  |
|  | Affective disorder | F30–F34, F38, F39 |
|  | Anxiety and behavioral disorder | F40–F45, F48, F50, F55, F59–F66, F68, F69 |
|  | Alcohol and drug abuse | F10–F19, Z50.2, Z50.3, Z71.4, Z71.5 |
|  | Schizophrenia | F20–F22, F25, F28, F29 |
